# Wegener's Granulomatosis: A Rare Cause of Hydronephrosis

**DOI:** 10.1155/2011/814794

**Published:** 2011-03-31

**Authors:** Julien Lillaz, Stéphane Bernardini, Marie-Paule Algros, Hugues Bittard, François Kleinclauss

**Affiliations:** ^1^Department of Urology and Renal Transplantation, University Hospital Saint Jacques 2, Place Saint Jacques, 25030 Besançon, France; ^2^University of Franche-Comté, 25000 Besançon, France; ^3^Department of Pathology, University Hospital Jean Minjoz, 25020 Besançon, France; ^4^INSERM U645, IFR 133, 25020 Besançon, France

## Abstract

A seventy-one-year-old woman was hospitalized at our institution for a right-sided “renal colic” associated with an infectious background. Alithiasic ureterohydronephrosis was diagnosed by imaging. A urinary diversion was thus performed using a double J endoureteral stent. The etiologic assessment of the hydronephrosis showed the presence of a periureteral mass that caused extrinsic ureteral compression. After surgical excision of the ureteral lesion, the Wegener's granulomatosis diagnosis was established. This report is the clinical description of a case of “atypical” Wegener's granulomatosis revealed by the onset of a ureteral disease mimicking a neoplastic process.

## 1. Introduction

Various etiologies of alithiasic hydronephrosis are known, the most frequent being of neoplastic origin. Obviously, retroperitoneal fibrosis and various systemic diseases should also be considered. Wegener's granulomatosis rarely affects the urological system. This case report discusses the case of a female patient in whom ureteral stenosis revealed Wegener's granulomatosis.

## 2. Case History

A 71-year-old female patient presented with an episode of right renal colic associated with an infectious syndrome. A few days before admission, she had shown signs of anorexia, loss of weight, and asthenia. At admittance, she showed a biological inflammatory syndrome (C reactive protein at 176 mg/L) with renal function alteration (creatinine clearance calculated by the Cockroft method: 51 mL/min/m^2^). Her baseline creatinine was not known. 

Imaging showed a right renal pelvic dilatation without any obstacle or visible extrinsic compression. A urinary diversion was performed using an endoureteral stent, and a double intravenous antibiotherapy was started. Perioperative retrograde pyelography showed a stenosis located at the level of the iliac ureter with an upper-ureteral dilatation but no endoluminal mass.

**Figure 1:**
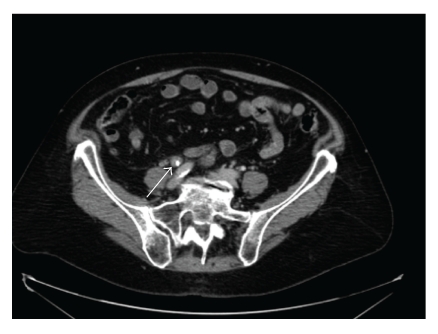
Abdominal computerized tomography performed after urinary diversion by ureteral stent. The figure shows a right iliac ureteral mass (white arrow) below the right iliac artery cross.

**Figure 2:**
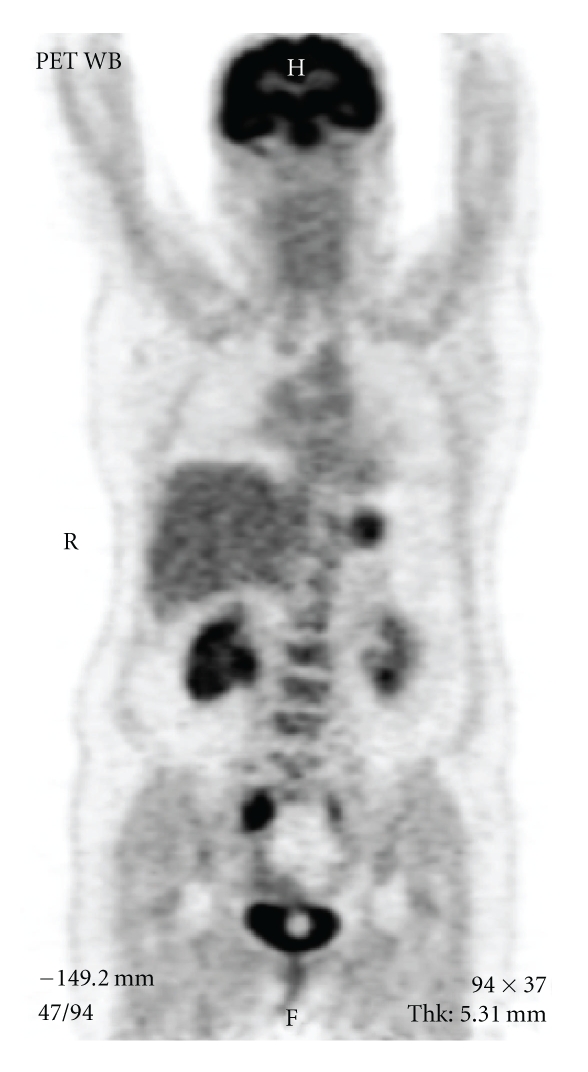
Positron emission tomodensitometry (PET) scan. A high fixation level on the right ureter and in the ENT area can be observed.

The treatment led to an increase in creatinine clearance, but the chronic fever and biological inflammatory syndrome persisted. At this stage, the bacteriological urine and blood samples had excluded urinary sepsis. A computerized tomography urography was then carried out. It showed a periureteral infiltration involving iliac vessels (Figure 1). Urine cytology showed nonspecific inflammatory and dystrophic cells but did not confirm the presence of a transitional cell carcinoma. The urinary sediment showed microscopic hematuria and proteinuria. The X-ray and computerized tomography of the patient's chest were normal. This led us to carry out a flexible ureterorenoscopy, which revealed an intraluminal mass. The biopsy of this mass showed inflammatory tissues but no transitional cell carcinoma. A PET scan was then performed. High fixation levels were found in the right ureter, ENT area, left lung, L4-L5 vertebra and spleen (Figure 2), which caused us to suspect lymphoma. This hypothesis was, however, ruled out by complementary tests. The clinical examination of the ENT area was normal. 

A sudden degradation of the renal function occurred and, at the same time, the creatinine level rose up to 240 *μ*mol/L, although there was no pelvic obstruction. We finally decided to perform a surgical exploration using the retroperitoneal approach. During the surgery, fibrotic tissue was found around the right ureter and iliac vessels. A segmental resection of the right ureter was performed. The pathological examination of the frozen section did not reveal any tumoral tissue. 

**Figure 3:**
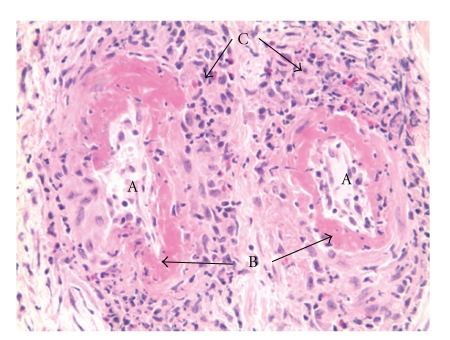
Pathological findings of the resected right ureter (Hematoxylin-eosin stain, ×20) As can be seen, two vessels (A) have thick fibrinoid deposits within their walls (B), sign of necrotic vasculitis. In the periphery, a dense polymorphous inflammatory infiltrate with polynuclear eosinophils (C) can be observed as well as granulomas.

The postoperative course was uneventful despite a stagnation of the renal function. A renal biopsy was then conducted. The final pathological examination of the resected ureter was concluded with the absence of neoplastic cells but brought to light a vasculitis aspect and necrosis (Figure 3). The renal biopsy confirmed these results and uncovered an extracapillary glomerulonephritis. Antineutrophil cytoplasmic antibodies (ANCAs) were found positive at a level of 1/80 while the level of PR3 was 38 UR/mL (normal <3.5 UR/mL). This confirmed the final diagnosis of Wegener's granulomatosis.

The patient was transferred to the Department of Nephrology for optimal treatment. She underwent steroid and cyclophosphamide therapies, which immediately improved her renal function and global status (serum creatinine: 166 *μ*mol/L at 6 months).

## 3. Discussion

 Wegener's granulomatosis is a vasculitis with a prevalence of around 1/42000. Both genders are affected, and the mean age at onset is within the fourth decade. It mainly affects the upper and lower respiratory tracts (including the otorhinolaryngologcal area) and the kidneys, leading to renal failure. It may also impact other organs like the skin or peripheral nerves, but it rarely involves the urological area. Nevertheless, Wegener's granulomatosis may affect large blood vessels such as the aorta or vena cava. Ureters may also be compressed by the large inflammatory mass. Rich and Piering describe a case of Wegener's disease relapse with ureteral compression that occurred after a renal transplantation [1]. Ureteral lesions secondary to Wegener's granulomatosis are very rare. In the literature there are about twenty documented cases with ureteral stenosis. The pelvic and iliac ureters are the main locations but some cases of multilevel and bilateral lesions have also been described [2, 3]. In patients with advanced Wegener's disease who suffer from bilateral compression, acute renal failure with anuria can be observed [4]. Other related systemic diseases may be complicated by ureteral stenosis: these are, for instance, the Churg Strauss syndrome [5], purpura rheumatica [6], dermatomyositis [7] and Kawasaki's disease [8]. 

The Wegener's granulomatosis case described in this paper was particularly difficult to diagnose. In our observation, the main and first symptoms of the disease were ureteral stenosis, renal colic with renal failure associated with an inflammatory syndrome. Even if the PET scan was able to locate the areas where Wegener's disease developed, the patient was completely asymptomatic in the lung and ENT areas. The chest and ENT imaging was normal. The results of the urological evaluation and PET scan, as well as the periureteral aspect, caused us to suspect a diagnosis of transitional cell carcinoma of the upper urinary tract. Moreover, this diagnosis could explain the inflammatory symptom and the alteration of the patient's performance status. 

To our knowledge, only Kamar et al. have already published literature on two patients with Wegener's granulomatosis exhibiting isolated ureteral stenosis [9]. The first presented with a high-degree fever, asthenia, arthralgia, and myalgia. The CT scan demonstrated unilateral hydronephrosis without any urinary symptom. The second was a woman with asthenia, low urinary tract infection, and lower back pain. An intravenous urogram showed unilateral hydronephrosis. In both cases, the ureteral stenosis was excised by open surgery. Histologic examination revealed lesions of Wegener's granulomatosis. 

Our case is similar, and describes a very particular aspect of Wegener's granulomatosis with isolated initial ureteral symptoms. The first suspected diagnosis was a transitional cell carcinoma of the ureter, and most of the tests were performed to confirm this diagnosis. After the surgical resection of the right ureter, however, a Wegener's granulomatosis diagnosis was established, and the patient was quickly referred to a specialized department where treatment was rapidly started.

## 4. Conclusion

Among the multiple causes of ureteral stenosis, the most frequent are the urologic neoplastic, or “extra urologic”, diseases. Wegener's granulomatosis may occasionally involve the urologic area. Our report shows that urologic lesions may reveal the disease. In the case where no other organ is affected, the diagnosis is then difficult to establish. In terms of prognosis, however, any ureteral mass should be considered as an upper urinary tract transitional cell carcinoma until the final pathological diagnosis is reached. 

## References

[1] Rich LM, Piering WF (1994). Ureteral stenosis due to recurrent Wegener’s granulomatosis after kidney transplantation. *Journal of the American Society of Nephrology*.

[2] Ben Ghorbel I, Chebbi W, Zouari M, Hentati F, Miled M, Houman MH (2006). Ureteral stenosis in Wegener's granulomatosis. Case report. *Presse Medicale*.

[3] Adelizzi RA, Shockley FK, Pietras JR (1986). Wegener’s granulomatosis with ureteric obstruction. *Journal of Rheumatology*.

[4] Le Thi Huong D, De Gennes C, Wechsler B, Etienne SD, Godeau P (1988). Anuria caused by bilateral ureteral stenosis in Wegener's granulomatosis. *Presse Medicale*.

[5] Azar N, Guillevin L, Huong Du LT, Herreman G, Meyrier A, Godeau P (1989). Symptomatic urogenital manifestations of polyarteritis nodosa and Churg-Strauss angiitis: analysis of 8 of 165 patients. *Journal of Urology*.

[6] Ihoriya C, Morita Y, Tokura T (2008). Bilateral ureteral stenosis as a complication of Henoch-Schonlein vasculitis. *Modern Rheumatology*.

[7] Morita Y, Sakuta T, Nagasu H (2007). Bilateral ureteral stenosis and duodenal perforation in a patient with dermatomyositis. *Modern Rheumatology*.

[8] Subramaniam R, Lama T, Chong CY (2004). Pelviureteric junction obstruction as sequelae of Kawasaki disease. *Pediatric Surgery International*.

[9] Kamar N, Malavaud B, Alric L (2003). Ureteral stenosis as the sole manifestation of Wegener’s granulomatosis. *Urology*.

